# Growth and cellular patterning during fetal human inner ear development studied by a correlative imaging approach

**DOI:** 10.1186/s12861-019-0191-y

**Published:** 2019-05-20

**Authors:** Lejo Johnson Chacko, David Wertjanz, Consolato Sergi, Jozsef Dudas, Natalie Fischer, Theresa Eberharter, Romed Hoermann, Rudolf Glueckert, Helga Fritsch, Helge Rask-Andersen, Anneliese Schrott-Fischer, Stephan Handschuh

**Affiliations:** Department of Otorhinolaryngology, Medical University Innsbruck, Anichstrasse 35, 6020 Innsbruck, Austria

## Abstract

**Background:**

Progressive transformation of the otic placode into the functional inner ear during gestational development in humans leads to the acquisition of hearing perception via the cochlea and balance and spatial orientation via the vestibular organ.

**Results:**

Using a correlative approach involving micro-computerized tomography (micro-CT), transmission electron microscopy and histological techniques we were able to examine both the morphological and cellular changes associated with human inner ear development. Such an evaluation allowed for the examination of 3D geometry with high spatial and temporal resolution. In concert with gestational progression and growth of the cochlear duct, an increase in the distance between some of the Crista ampullaris is evident in all the specimens examined from GW12 to GW36. A parallel increase in the distances between the macular organs - fetal utricle and saccule - is also evident across the gestational stages examined. The distances between both the utricle and saccule to the three cristae ampullares also increased across the stages examined. A gradient in hair cell differentiation is apparent from apex to base of the fetal cochlea even at GW14.

**Conclusion:**

We present structural information on human inner ear development across multiple levels of biological organization, including gross-morphology of the inner ear, cellular and subcellular details of hearing and vestibular organs, as well as ultrastructural details in the developing sensory epithelia. This enabled the gathering of detailed information regarding morphometric changes as well in realizing the complex developmental patterns of the human inner ear. We were able to quantify the volumetric and linear aspects of selected gestational inner ear specimens enabling a better understanding of the cellular changes across the fetal gestational timeline. Moreover, these data could serve as a reference for better understanding disorders that arise during inner ear development.

**Electronic supplementary material:**

The online version of this article (10.1186/s12861-019-0191-y) contains supplementary material, which is available to authorized users.

## Background

The sense organs for hearing and balance - the cochlea and the vestibular organ - are located in the temporal bone. Inner ear development initiates from the ectoderm, which in humans thickens to form the otic placode by gestational week (GW) 4 [1]. The otic placode develops into the primordium, which in turn forms the otic vesicle [2]. Further proliferation grounds the development of the primordial endolymphatic labyrinth and the cochlea. The developing membranous labyrinth contains the immature sensory epithelia which later differentiate and form the hair cells and the supporting cells. The afferent flow of information transmitted towards the brain from these hair cells occurs via neurons enclosed by Schwann cells and satellite glial cells. With the exception of Schwann cells [3], satellite glia, strial intermediate cells (of neural crest origin) [4] and potential cells belonging to the reticulo-endothelial system (RES) all the other cell types of the inner ear are derivatives of the otic placode.

Information on the fetal human inner ear development is not yet complete. The gestational development has been subdivided into two distinct phases termed the embryo and the fetus. The period immediately after fertilization until GW8 is termed the embryonic phase of human development. The fetal phase of human development starts with GW9 and continues onwards until birth. Characterization of human developmental stages, both during embryonic and fetal development, is assessed using Crown Rump Length (CRL) [5]. Awareness of CRL measurement can be used to identify anomalies in development like underdevelopment or growth indicative of an underlying disorder [6].

Cellular differentiation and morphogenesis cause the otic placode to form the otic vesicle, which later forms the primordial endolymphatic labyrinth and the cochlea. Several transcriptional factors and developmental genes, many of which are expressed in cohesion, enable this cascade of morphological changes. Prime among these cellular patterning genes are Maf B (V-maf musculo-aponeurotic fibrosarcoma oncogene homolog B) and Pax family genes including Pax2, Pax6, and Pax8. Besides these patterning genes, other genes specific for the neurosensory epithelia are also functional at this time point including peripherin and Class III β-Tubulin [7] whose expression are guided by neurotrophins and their factors [8]. Neurosensory cell fate specification [9] is driven by these and other factors and thus leads to the growth of a well-designed sensory organ capable of auditory and vestibular cognizance.

Previous research has revealed a multiple increase in labyrinthine geometry between GW9 and GW18 after which it remains relatively stable. This stabilization of labyrinthine length could be accounted for by the ossification of the otic capsule layer around the fetal inner ear starting at GW21 [10]. It is around this timespan vestibular (GW17) [11] and cochlear function (GW19) are reached [12]. However, the superior and posterior semicircular canals (SCC) do not reach adult dimensions before GW25. The cochlea attains near adult size by GW23. However, the differentiation process of the organ of Corti (OC) is completed by GW30 [13].

Although the cochlear development lags behind the vestibulum, its morphogenesis is no less complex. The cochlear duct contains two sensory domains: the greater epithelial ridge (GER) with the Kölliker’s organ (KO) [made up of columnar epithelial supporting cells] on the medial side and the lesser epithelial ridge (LER) on the lateral side. Around GW10 a single row of inner hair cells (IHC) starts to develop in the GER [14] while three rows of outer hair cells (OHC) replaces the LER. Previous studies have reported that by GW13 the KO is entirely replaced by supporting and IHC’s [15] at the basal turn of the cochlea.

Currently available data regarding changes in the length and volume of the cochlear duct in human fetuses dates back to the 1960’s. In a study by Engström and co-workers, fetal specimens were fixed with 1.5% osmic acid solution, followed by extraction of the petrous part of the temporal bone [16]. A “surface specimen” technique enabled the utilization of these inner ears for histology. Cochlear duct/scala media length increased from 20 mm at three months (GW12) to a maximum of 39.8 mm at GW24. Additionally, an increase in the number of IHCs and OHCs throughout these gestational weeks was demonstrated. At GW12 kinocilia are present on sensory cells of the organ of Corti as well as on supporting cells [17] while in the mature OC, kinocilia are absent.

Although the human fetal cochlea has not been examined in detail, the contrary is true for the adult human cochlea, which coils up 2.25 to 3 times. The length of the scala media in the adult human cochlea has been examined in several studies. The earliest histological measurements by Gustaf Retzius revealed a mean length of 33.5 mm while more recent studies performed using Synchrotron Radiation Phase-Contrast Imaging reveal a mean scale media length of 32.5 mm [18]. There are significant interspecies variations in cochlea length and the number of cochlear turns. In 1884, Retzius described that the pattern of sensory cells in the human organ of Corti lacks the geometric regularity often seen in lower mammals, which has been substantiated in more recent electron micrographic studies [19]. All these factors make it imperative to examine the development of the human fetal cochlea in more detail.

Estimation of developmental progress of fetal inner ear specimens requires histological processing or usage of non-destructive imaging techniques [20]. Detailed data currently exists on the human embryonic inner ear between the Carnegie Stages (CS) 12 (GW4) to 23 (GW8) using X-ray micro-tomography (micro-CT) [1]. Other techniques like synchrotron radiation-based micro-computed tomography are useful in examining excised human temporal bones [21–23]. Micro-CT based techniques have been proven to be useful in rendering and measuring the geometry of the human SCC’s [24]. We have previously evaluated the same technique in excised adult human temporal specimens [24, 25].

Animal developmental models in inner ear research have frequently used mice, chick, zebrafish, rats and guinea pigs. The gestational period of mice lasts 16 days, and hearing ability initiates at postnatal day 16. The developmental progression of the murine inner ear has been examined previously using thin sheet laser imaging microscopy (sTSLIM) [26]. This study is of particular interest, as it demonstrates the developmental progression of the murine inner ear model. The scala media in these mice showed an overall increase in length and volume throughout gestation.

To acquire a deeper and more profound understanding of the development of the fetal human inner ear, a correlative approach using both non-destructive 3D imaging and 2D histological techniques are essential. Such methods could overcome limitations of traditional and singular approaches and would enable assessement of inner ear features while avoiding artifacts. The present study aims to elucidate the different developmental stages in non-destructive 3D micro-CT scans combines with histological techniques in order to evaluate selected regions of interest. Semi-thin sections from selected areas could be compared with micro-CT scans, which could also be combined with EM imaging.

## Results

The development of the human cochlea starts with the elongation of the anterior end of the otic vesicle to form the cochlear duct. This cochlear duct is the precursor of the endolymph-filled scala media, which houses the organ of Corti. Micro-CT imaging of excised human fetal temporal bones followed by manual segmentation enables 3D rendering of the imaged datasets. Based on image segmentation we measured the length and volume of the fetal cochlear duct. Subsequently, the same specimens were then used for histological sectioning (primarily semi-thin sectioning) and then for electron microscopic evaluation of select regions of interest.

**Fig. 1:**
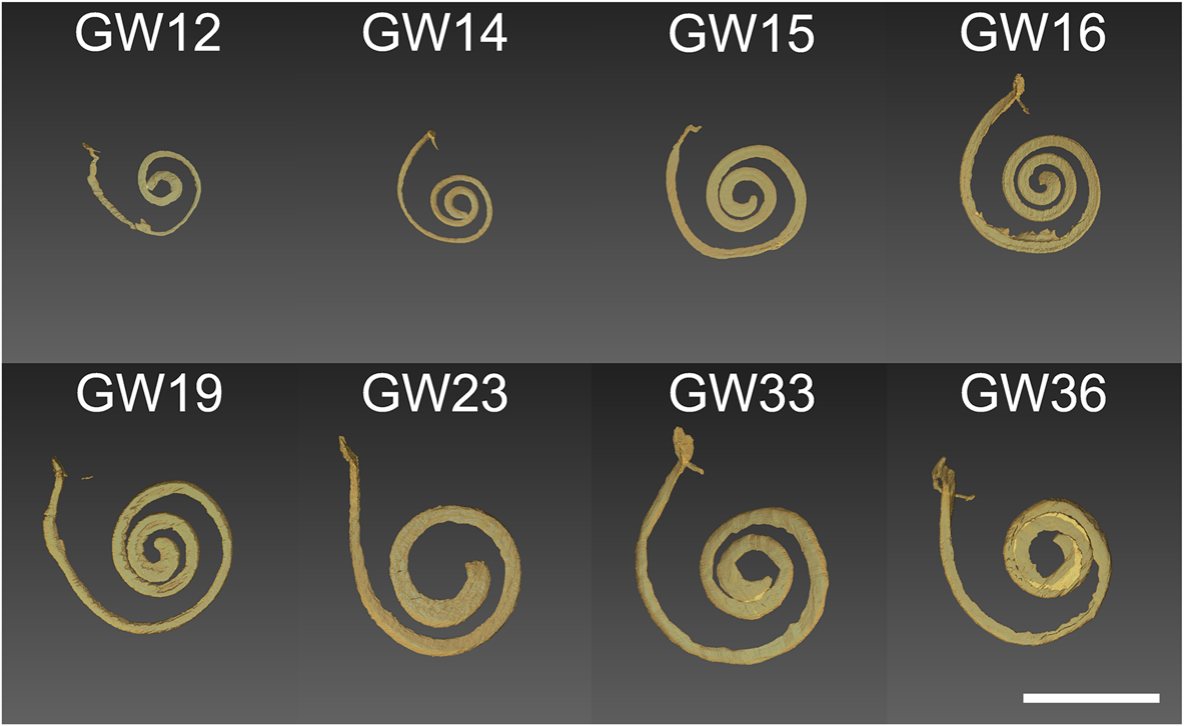
Growth of the cochlear duct/ scala media between the gestational weeks 11 to 36. 3D images are manually segmented fetal human temporal bones rendered using Amira 6.4® software. Substantial increase in the endolymphatic fluid volume and length of the scala media were apparent in the segmented datasets. Since all specimens were randomly oriented during scanning all the left datasets (GW14, GW19, GW23 & GW36) were mirrored so that all the specimens appear to be that of a right inner ear

**Fig. 2:**
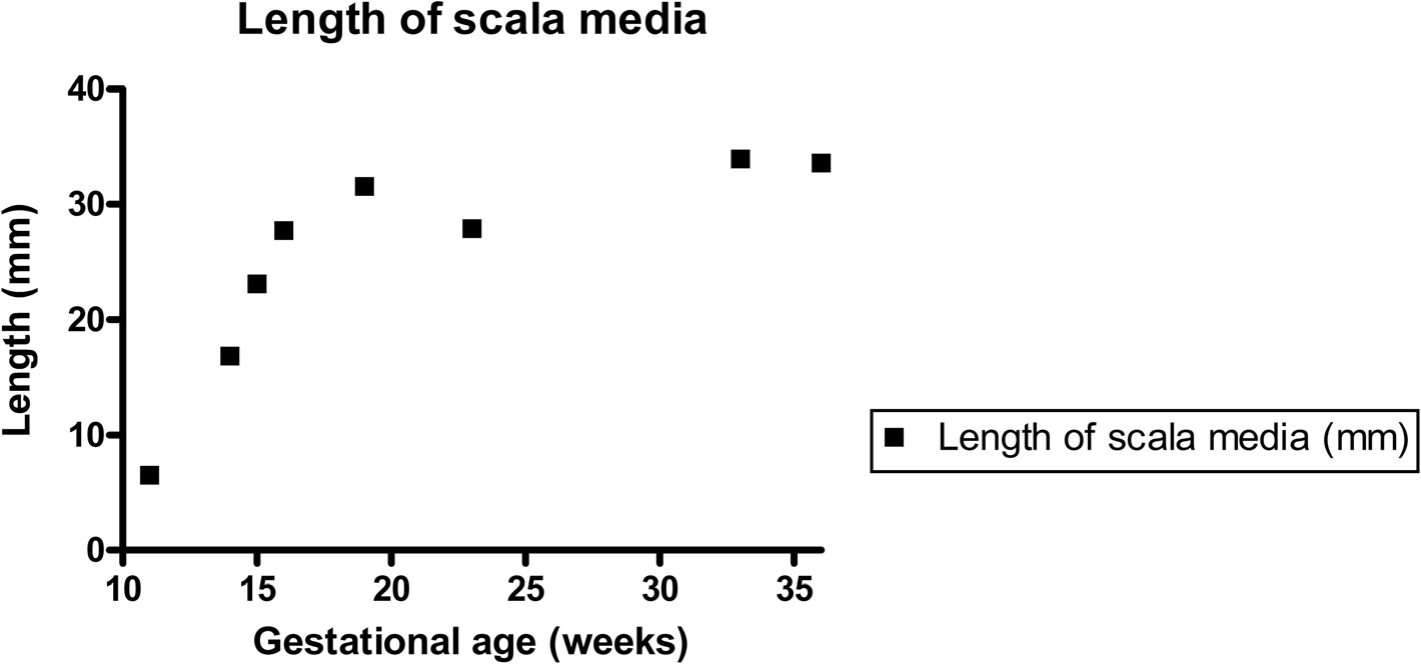
Change in the length of the scala media between the gestational weeks 11 to 36. The growth of the endolymph filled scala media estimated via centerline analysis using Amira 6.4®. A substantial increase in scala media length measured from apex to base occurs between the gestational age 11 (youngest specimen examined) and gestational age 36 (oldest specimen examined), while strongest increase in analyzed stages occurs between GW 11 and GW19

**Fig. 3:**
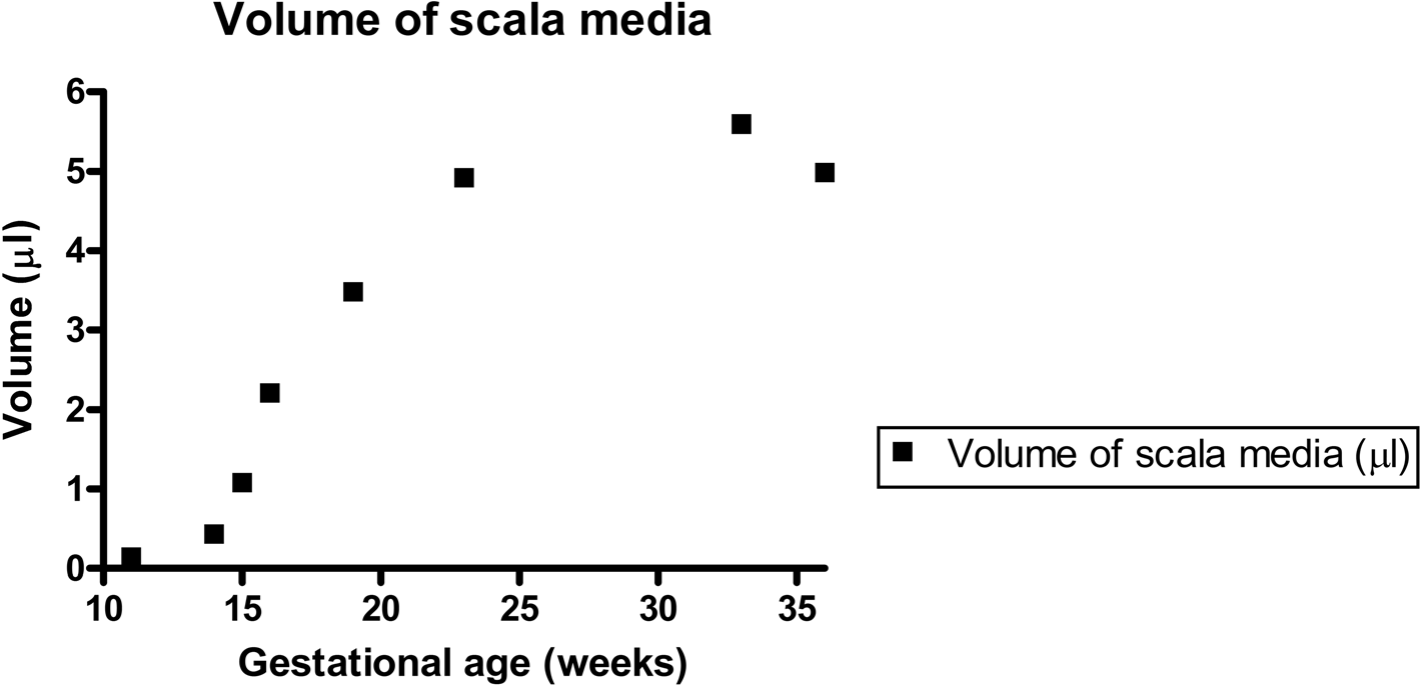
Change in the volume of the scala media between the gestational weeks 11 to 36. The increase in volume of the endolymph filled scala media estimated via material statistics module using Amira 6.4®. A substantial increase in scala media volume measured occurs between the gestational age 11 (youngest specimen examined) and gestational age 36 (oldest specimen examined), while strongest increase in analyzed stages occurs between GW 14 and GW23

**Fig. 4:**
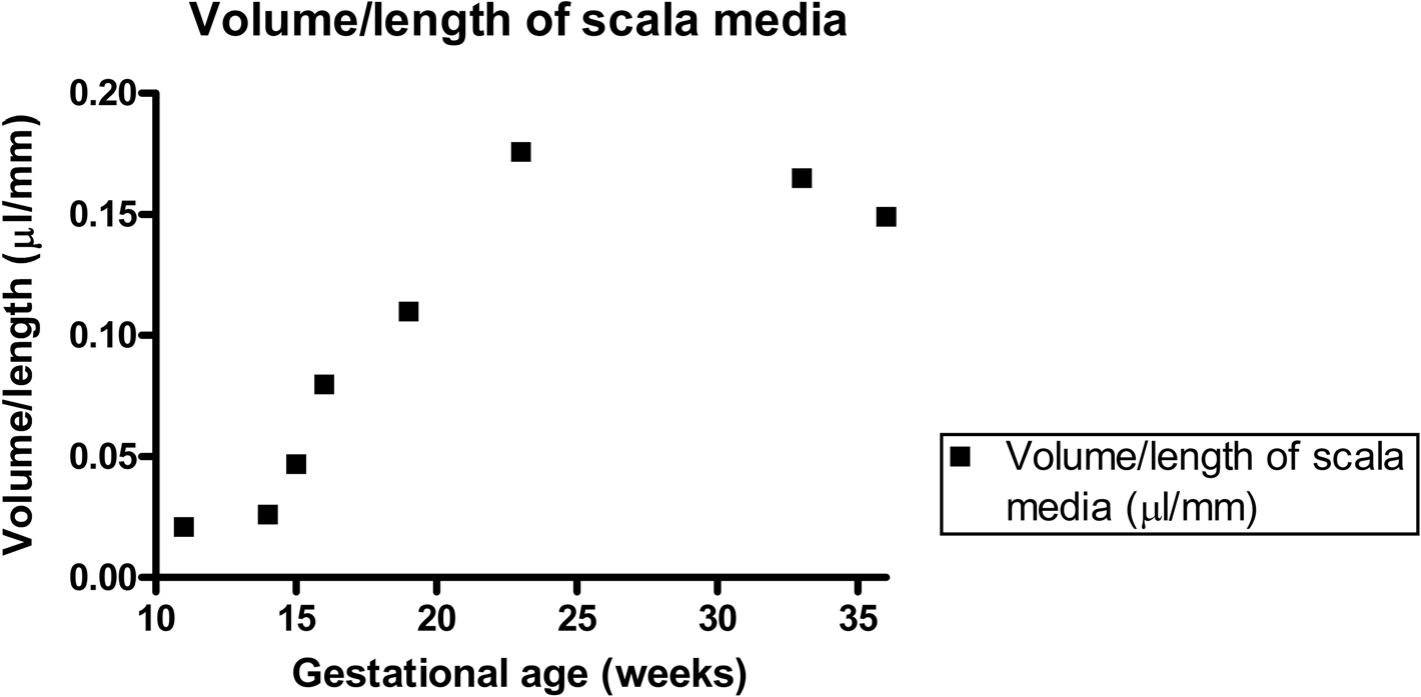
Change in volume/length ratio of the scala media between the gestational weeks 11 to 36. The increase in the volume/length ratio of the scala media parallels the increase in the volumes of the scala media of individual fetal specimens. A substantial increase in this ratio occurs between the gestational ages 11 (youngest specimen examined) and gestational age 23

Examination of the cochlear duct/ scala media demonstrated a gradual but consistent increase in the length of the cochlear duct throughout the stages examined (GW11 until GW36, Fig. 1). This increase in length was continuous for all the gestational specimens tested with the only exception being for the GW23 sample. Strongest increase in scala media length occurred from GW11 to GW 19, while only little increase in length occurred after GW19 (Fig. 2). In parallel, the volume of the cochlear duct/ scala media also increased as gestational age-progressed until GW33 following which it declines. Strongest increase in scala media volume occurred between GW14 and GW23 (Fig. 3). In concert with the increase in the volume and length of the cochlear duct, the ratio between these two parameters (Fig. 4) also increased until GW23 following which the ratio dropped.

**Fig. 5:**
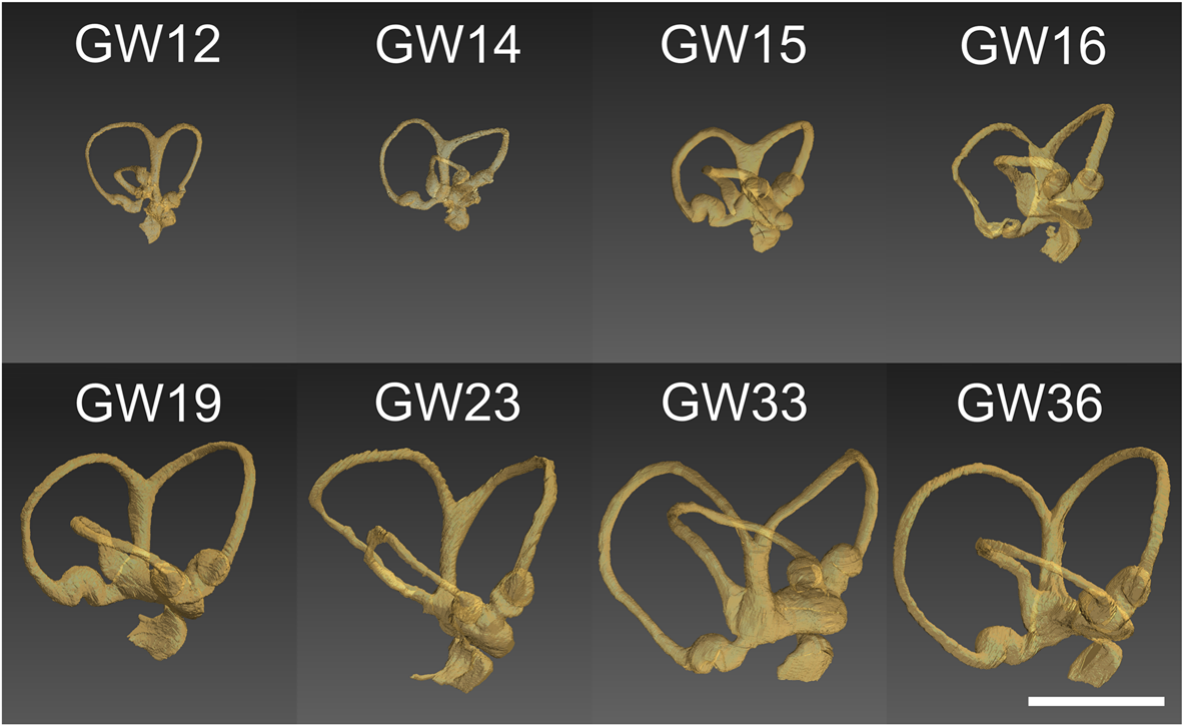
Growth of the endolymph between the gestational weeks 11 to 36. 3D rendered images obtained from manually segmented fetal human temporal bones using Amira 6.4® software. Substantial increase in the endolymphatic fluid spaces was visually apparent in the segmented datasets. Since all the specimens were randomly oriented during scanning all the left datasets (GW14, GW19, GW23 & GW36) were mirrored so that all the specimens appear to be that of a right inner ear

**Fig. 6:**
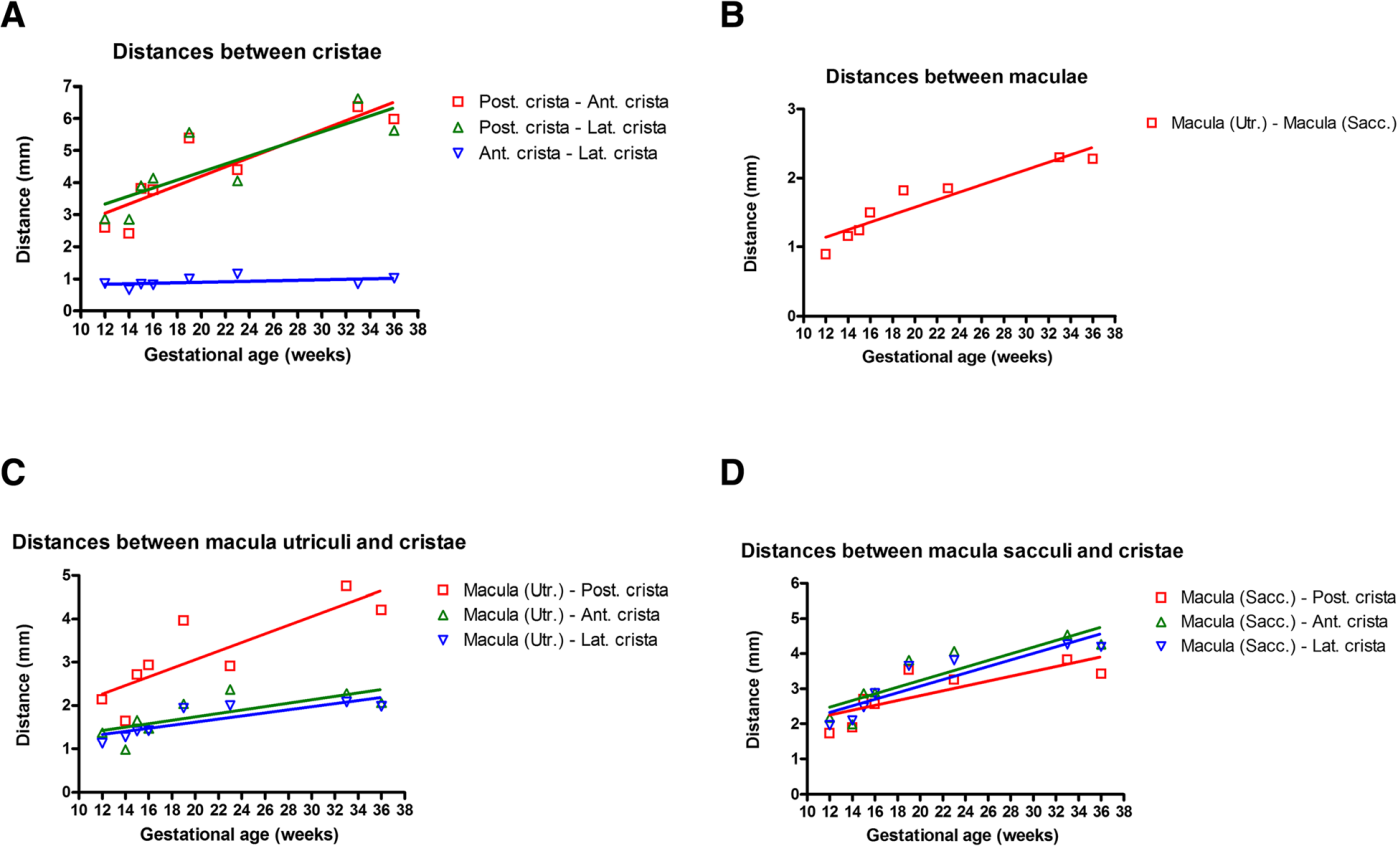
Variation in the distances between the vestibular end organs during the gestational weeks 11 to 36. Trend lines added based on linear regression. **a** Change in distance between the developing cristae ampullares. **b** Change in distance between the developing macula organs. **c** Change in distance between the developing macula of the utricle and the three individual cristae ampullares. **d** Change in distances amongst the developing macula of the saccule and the three individual Cristae ampullares between the fetal gestational weeks 11 to 36

In concert with gestational progression, an increase in the endolymphatic fluid spaces occurred (Fig. 5). A substantial increase in the distance between some of the Crista ampullaris is also evident. The distance between the posterior Crista ampullaris to the anterior crista ampullaris and posterior to the lateral crista ampullaris increases in all the specimens examined from GW12 to GW36 (Fig. 6a). However, the distance between the anterior Crista ampullaris to the lateral crista ampullaris remains more or less constant across the gestational ages examined. Meanwhile, a parallel increase in the distances between the macular organs -fetal utricle and the fetal saccule - is also evident across the gestational stages examined (Fig. 6b). In parallel the distances between the fetal utricle to the vestibular end organs (Fig. 6c) also increases across all the gestational ages examined. The increase in length is highest between the utricle to the posterior Crista ampullaris in all the gestational ages examined. The length between the utricle to the anterior Crista ampullaris and the lateral Crista ampullaris is much shorter although the distance increases slightly as the gestational age progresses. The distance between the macula of the saccule (Fig. 6d) which is furthest away from the three cristae ampullares also increases with gestational progression. The anterior Crista ampullaris is furthest away from the macula of the saccule while the posterior Crista ampullaris is the closest. The distance between the macula of the saccule and the three cristae almost doubles in size between GW12 (earliest stage examined) and GW36 (oldest stage examined).

**Fig. 7:**
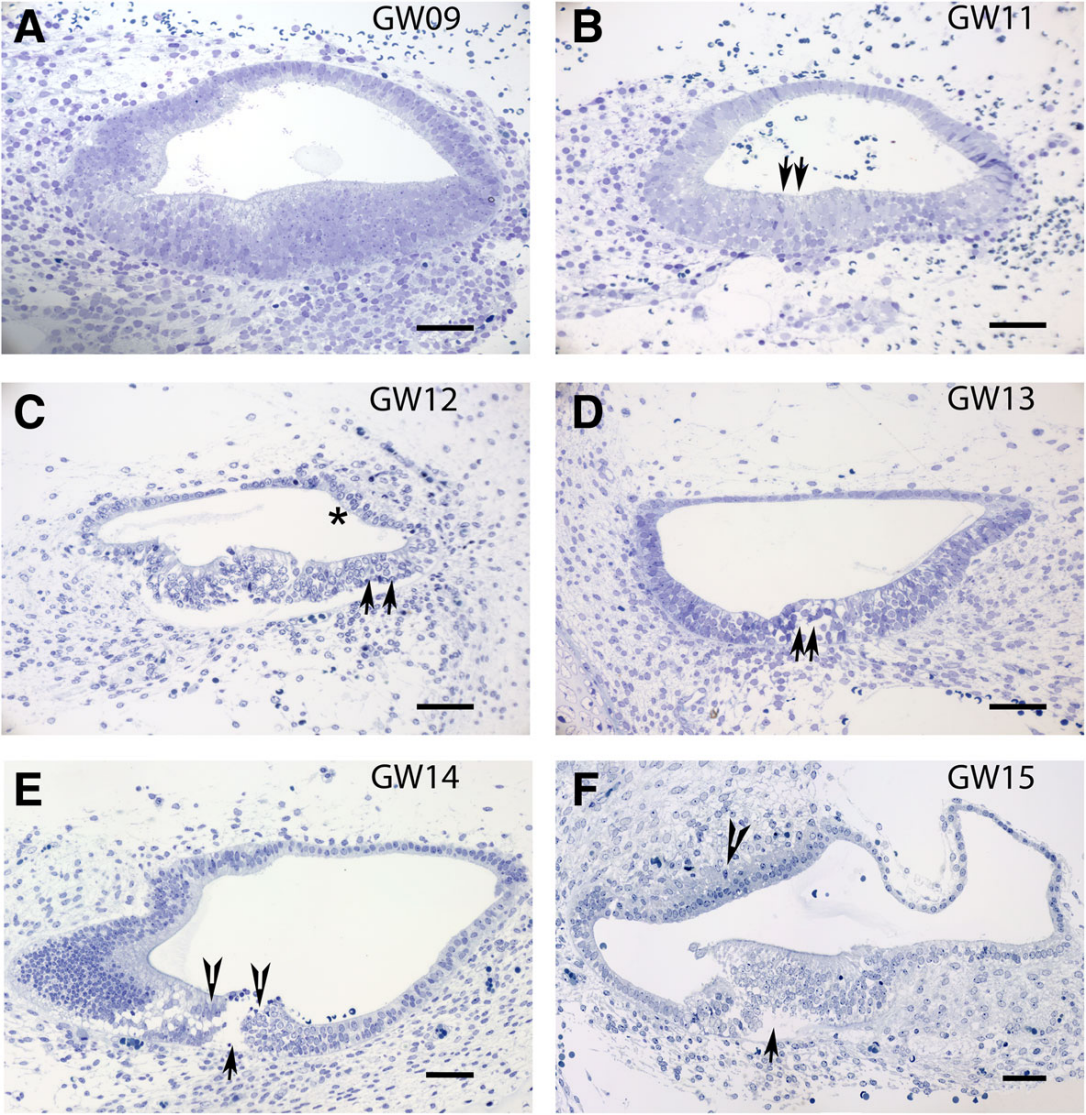
Overview of the developing fetal inner ear from GW9 to GW15 along the basal turn. **a** Toluidine blue stained semi-thin section of a fetal human cochlea at GW9 showing the basal turn. Accumulation of epithelial cells is evident at this stage, but no differentiation in the supporting cells or hair cells is evident. **b** Toluidine blue stained semi-thin section of a fetal human cochlea at GW11 showing the basal turn. At this gestational age, supporting cells (arrows) show distinct morphological characteristics. **c** Toluidine blue stained semi-thin section of a fetal human cochlea at GW12 with the stria vascularis (asterisk) and supporting cells (arrows) evident. **d** Toluidine blue stained semi-thin section of a fetal human cochlea at GW13 with tunnel of Corti development starting. The developing outer and inner hair cells are also apparent (arrow) in the histological section. **e** Toluidine blue stained semi-thin section of a fetal human cochlea at GW14. The opening of the tunnel of Corti (arrow) is evident at GW14 with the outer and inner hair cells lining the developing OC being apparent (arrowheads). **f** Toluidine blue stained semi-thin section of a fetal human cochlea at GW15 the tunnel of Corti is enlarged (arrowhead). The cytoarchitecture of the stria vascularis (arrow) is well-developed. Melanocytes invading the basilar membrane in the future stria vascularis are also visible (arrowhead). Scale bars = 50 μm

Semi-thin sectioning of the fetal human inner ear reveals the accumulation of undifferentiated epithelial cells in the basal cochlea at the earliest gestational age examined (GW9) (Fig. 7a). At this point, the regions corresponding to the fetal Kölliker’s organ has not yet begun to show signs of the turnover process that would lead to the development of the fetal cochlear hair cells and the organ of Corti. Towards GW11 although the differentiation processes are still ongoing, the supporting cells (Fig. 7b arrows) underlying the base of the immature sensory epithelium has begun to show distinct morphological characteristics. By GW12 more supporting cells (Fig. 7c arrows) underlying the sensory epithelium as well as the epithelial lining [which later on forms the stria vascularis (Fig. 7c asterisk)] exhibits matured characteristics. Already a second cell layer exists invading from the spiral ligament probably forming the intermediate cells. By GW13 (Fig. 7d) the cochlear hair cells (arrows), as well as the tunnel of Corti, are apparent. By GW14 the tunnel of Corti (Fig. 7e arrow) has widened with the inner and outer hair cells (Fig. 7e arrowheads) being present. Towards GW15 the cytoarchitecture of the stria vascularis (Fig. 7f arrowhead) and the tunnel of Corti (Fig. 7f arrow) is more clearly present. At this stage the neural-crest-derived melanocytes (Fig. 7f arrowhead) migrate into the cochlea and penetrate into the basement membrane of the lateral wall epithelium which later forms the intermediate cells of the stria vascularis.

**Fig. 8:**
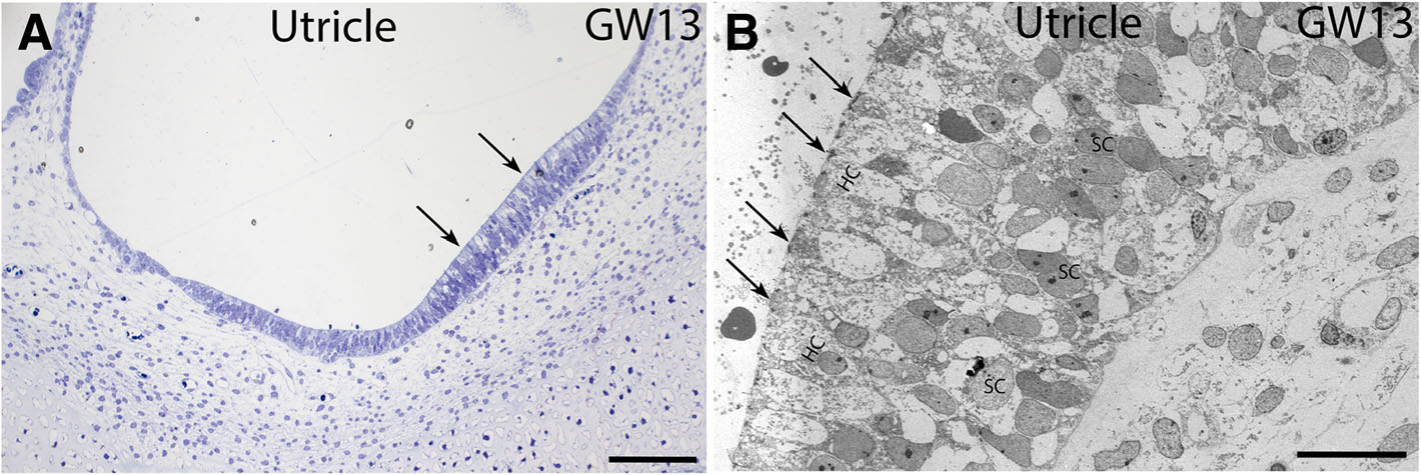
Correlative microscopy of the fetal human utricle at GW13. **a** Semi-thin section of the utricle at GW13. Hair cell formation clearly can be seen (arrows). Scale bar = 100 μm. **b** Transmission electron microscopy of the utricle at GW13. This figure demonstrates the hair cells (HC) with the cuticular plate, the stereocilia and the kinocilium (arrows). SC = supporting cells. Scale bar = 20 μm

Semi-thin sections also (Fig. 8a) revealed that developmental progression in the human fetal utricle is more advanced compared to the cellular differentiation in the organ of Corti. Vestibular hair cells are already lining the utricular sensory epithelia by GW13. Transmission electron microscopic imaging of the same specimen (Fig. 8b) reveals the utricular hair cells, their stereocilia as well as the underlying supporting cells during this stage of development. The correlative approach we pursued using microCT, semi-thin sectioning and transmission electron microscopy enabled the visualization of these utricular hair cells at GW13.

**Fig. 9:**
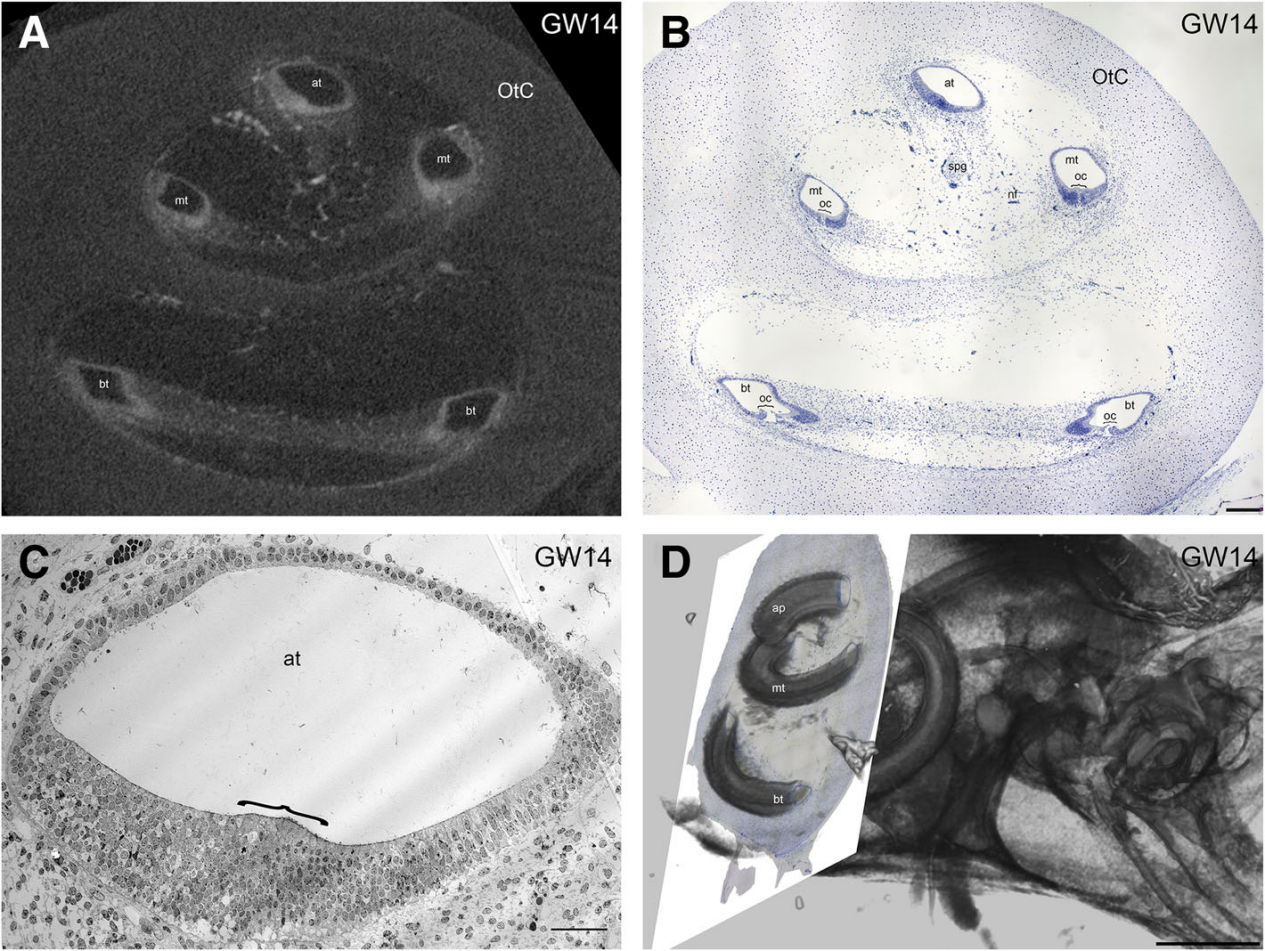
Correlative imaging of the human fetal cochlea at GW14 illustrating the basal to apical gradient in the developmental progression of the organ of Corti (oc) along the cochlear duct. **a** Mid-modiolar microCT section through the whole cochlea at GW14 showing basal turn (bt), middle turn (mt) and apical turn (at) of the cochlear duct. **b** Corresponding mid-modiolar semi-thin section of the same specimen. The opening of the tunnel of Corti in the basal turn is well visible (see also Fig. 7e), while in the apical turn the tunnel of Corti has yet to form. Along the middle turn, the opening of the tunnel of Corti is very small. Furthermore, the developing spiral ganglion (spg) and nerve fibers (nf) can be seen. **c** Transmission electron microscopy of the same specimen confirmed that in the apical turn the tunnel of Corti has not yet formed. d Volume rendering of the microCT scan showing the exact position of the registered semi-thin section depicted in Fig. 9b. Scale bar (**a**-**b**) 500 μm (**c**) 5 μm and (**d**) 1 mm


Examination of microCT slices (Fig. 9a) and semi-thin sections (Fig. 9b) from a GW14 specimen reveal a gradient in hair cell differentiation and organ of Corti formation. The organ of Corti is precisely formed in the basal turn while in the middle turn only differentiating cells, and the tunnel of Corti is apparent. Both the number of differentiating cells and the tunnel of Corti size in the middle turn is smaller when compared to the basal turn. There are no differentiating cells in the apical turn as apparent at the electron microscopic level (Fig. 9c) Registration of the semi-thin section onto a three dimensional rendering of the developing fetal inner ear reveals the disparity in the developmental process (Fig. 9d; Additional file 1: Video S1).

**Additional file 1: Video S1.** Correlative approach demonstrating a three-dimensional rendering from a microCT scan of a 14 gestational week old fetal temporal bone registered onto a light microscopic image of a histological section from the very same specimen. (MPG 8997 kb)


**Fig. 10:**
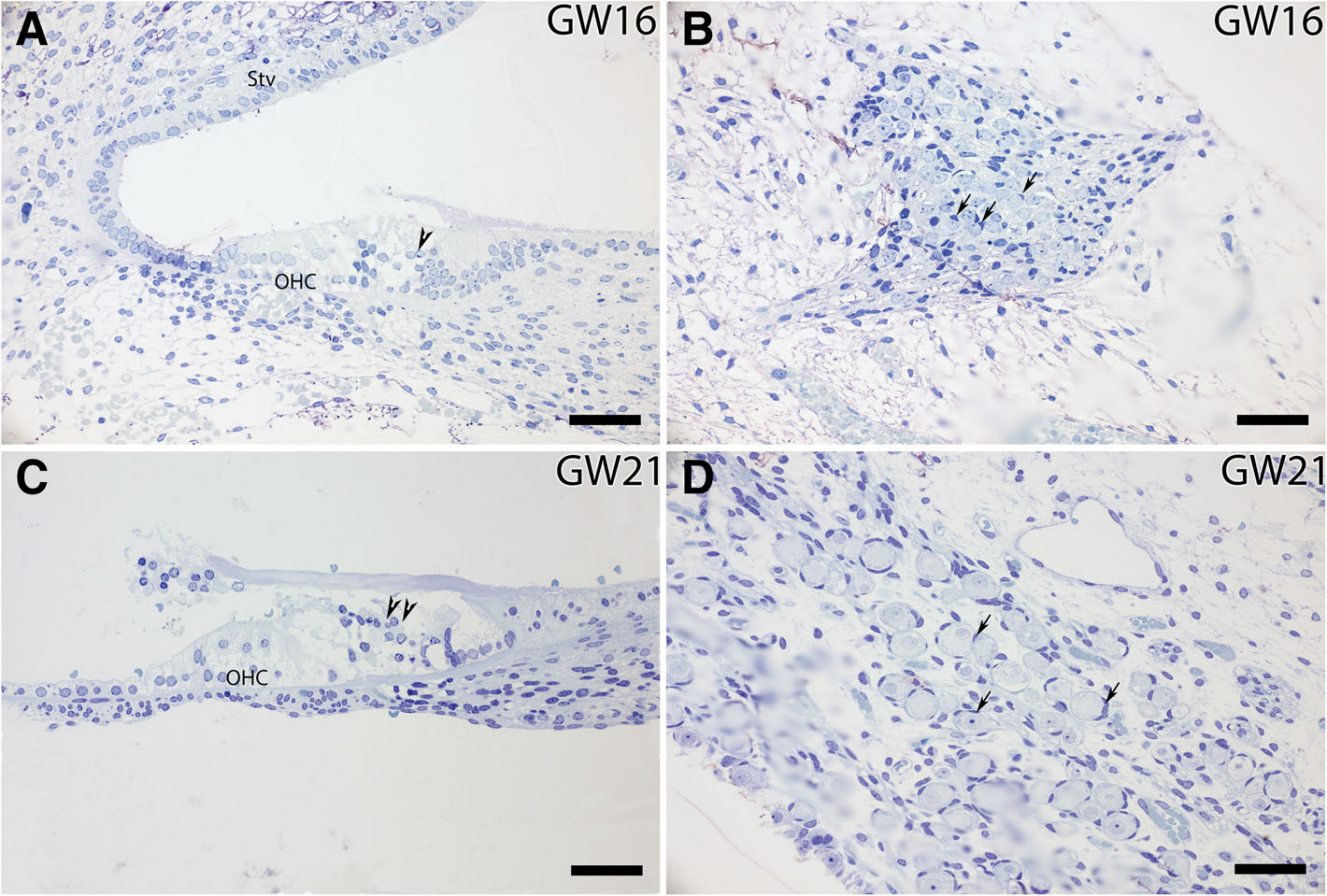
Semi-thin section of the developing OC. (**a**) At GW16, the organ of Corti has four rows of outer hair cells (OHC), inner hair cells (arrowhead) and the cytoarchitecture of the Stria vascularis (Stv) is well developed. **b** The spiral ganglion has a lone satellite glial cell (**b** arrow) encapsulating it at this stage. **c** At GW21, the organ of Corti (**c**) is well formed and functional with outer hair cells (OHC) and inner hair cells (arrowheads) visible. Three to four satellite glial cells (**d** open arrows) encapsulating the spiral ganglion cells (**d** arrows) is evident at this stage. Scale bars = 50 μm

With progressing gestational age, semi-thin sections from a GW16 specimen reveal the organ of Corti with four rows of OHCs placed on top of the future Deiters cells. Adjacent to the OHCs the cells of Hensen are already evidenced (Fig. 10a). The inner hair cells (arrowhead) are also apparent here. Also at this stage a few satellite glial cells (Fig. 10b) are formed around the spiral ganglion cells. By GW21, the cytoarchitecture of the organ of Corti (Fig. 10c) is nearly adult like with the outer hair cells (Fig. 10c OHC label) and inner hair cells (arrowheads) apparent. More of the satellite glial cells (Fig. 10d) 3 to 4 numbers are encapsulating the spiral ganglion cells at this stage of development.

**Fig. 11:**
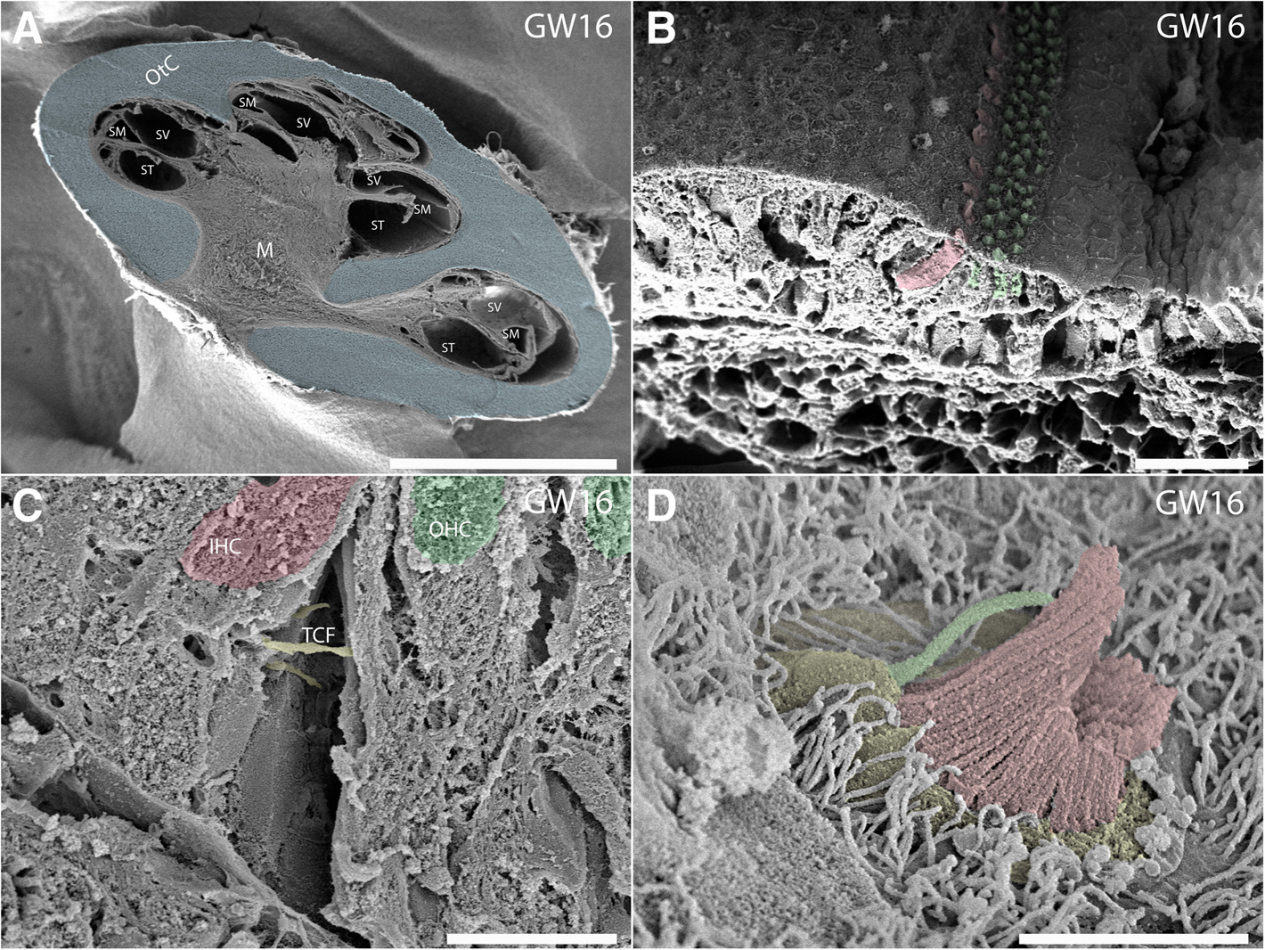
Scanning electron micrographs of a human cochlea at gestational week 16: **a** A mid-modiolar cut exposes the fluid compartments Scala media (SM), Scala tympani (ST) and Scala vestibuli (ST) that are all fully developed. Cartilaginous tissue is colored blue and borders the Modiolus (M). **b** Shows a detailed view of Kölliker’s organ of the middle turn with inner hair cells colored red and outer hair cells colored green. Note the primary cilium that is present in all supporting cells of the sensory epithelium! The basilar membrane is coated with a thick, spongiform layer of mesothelial cells. **c** The higher magnified view depicts the interior of the evolving tunnel of Corti. Medial efferent fibers span the gap between pillar cells as tunnel crossing fibers (TCF) to provide efferent innervation at the basal pole of outer hair cells (OHC, green). Inner hair cell (IHC) red. **d** The apical end of an inner hair cell is presented: The terminal web of the cuticular plate (colored yellow) is surrounded by long microvilli. Stereocilia (colored red) show typical arrangement similar to adult vestibular hair cell formation. A kinocilium (colored green) at the distal aspect of the apical hair cell pole is still present, its bulging insertion indicates the position of the basal body underneath. Scale bars (**a**) 4 mm, (**b-c**) 40 μm and (**d**) 4 μm

In addition to the correlative approach featuring microCT scans, semi-thin sectioning, and EM imaging, scanning electron microscopy was also performed using a GW16 specimen (Fig. 11). Scanning along the mid-modiolar plane of the fetal cochlea (Fig. 11a) reveals the developing cochlear ducts and is an indicator of the gradient in development. The organ of Corti and other supporting features are well developed in the basal and middle turns of the cochlea at this time point. One row of IHCs and 3–4 rows of OHCs are also evident at this time point along the middle turn of this specimen (Fig. 11b). The tunnel crossing fibers (TCF) traversing the tunnel of Corti horizontally are also apparent with other fibers progressing towards the tunnel spiral bundles (TSB) are present adjacent to it (Fig. 11c). Meanwhile the kinocilium (primary cilium) is still apparent adjacent to the stereocilia of the IHCs at GW16 in the middle turn of this specimen (Fig. 11d). The basal body of this primary cilium has integrated into the cuticular plate which encapsulates the base of the stereocilia. This primary cilium later degenerates leaving the basal body as a remnant. Microvilli emerging from the surrounding supporting cells are also apparent around the hair cells at this stage of development.

## Discussion

Hearing loss and vestibular disorders have a severely debilitating effect on humans with major causes being loss of hair cells or neural damage in spiral or vestibular ganglions. Intrinsic pathways guiding the development of the human inner ear could be utilized for rehabilitating the damaged adult human inner ear [27]. Currently there exists a dearth in the knowledge base regarding human inner ear development which is essential for the rejuvenation of the hair cells and spiral ganglion cells.

The inflammatory immune system composed of neurotrophic cytokines/chemokines guide the development of the most primitive stages of both sensory and neuronal development. The functional role of these moieties predate both brain-derived neurotrophic factors and glial growth factors in inner ear development and has been reported previously in frogs, mice and zebrafish [28]. Functional analyses of mutant mice models specific for members of the neurotrophin family have previously revealed malformations [29] but no evidence exists regarding changes in inner ear length and volume.

The human inner ear development starts in the embryo at GW4 and continues into the fetal stages of development. This process accelerates towards GW8 in the human fetus when the primeval Kölliker’s organ (precursor of the adult Organ of Corti) starts to differentiate. This differentiation process is guided by a multitude of regulatory molecules including BDNF [8]. Although early inner ear innervation is critically dependent on BDNF and Neurotrophin-3 expression [30] only BDNF and its associated receptors (p75NTR and Trk receptors) have been investigated so far in the fetal human inner ear [8]. Expression of BDNF and other progenitor molecules sets the stage for the attainment of hearing (GW19) [12] and balance (GW17) [11] functionality. Here we have examined the overall human inner ear development using micro-CT imaging, histological processing, and electron microscopy.

The spiral cochlear duct which later forms the Scala media contains the hearing receptor - the organ of Corti, which emerges quite early on during cochlear development. The other conduits- the Scala vestibuli and Scala tympani arise later on - developing both around and above it. Due to these factors, we chose to measure cochlear duct parameters such as length and volume as a reference to visualize and quantify inner ear growth during development. Although the earliest specimen we examined belonged to GW9, we were unable to fully render these due to the incomplete development of the cochlear duct in the said specimen.

Three-dimensional rendering of the segmented cochlear ducts revealed the continuous growth of the cochlear duct i.e. scala media between the gestational ages 11 to 36. A significant increase in the cochlear duct size occurs until GW19 following which the growth rate as evident from the length and volume measurements is much lowered. This spurt in scala media length and volume till GW19 could be accounted for by the rapid changes occurring in the cytoarchitecture before the beginning of hearing functionality at GW19 in the cochlea [12] and GW17 in the vestibulum [11]. Towards GW19 the scala media measured 31.56 mm in length and 3.48 μl in volume while at GW33 the length was at 33.94 mm and volume at 5.6 μl. Though the period between GW19 and GW36 showed only a gentle increase in scala media length, the scale media volume almost doubles. This increase in volume is accompanied by the morphological changes occurring in the developing cochlea as apparent from the semi-thin sections. The scala media length at GW33 was 33.94 mm and the volume at 5.6 μl while at GW36 the scala media length was at 33.60 mm and volume at 4.99 μl. This deviation observed could be a result of inter-subject variation. However the scala media volume we found at GW33 closely matches the average scala media volume 5.67 μl recorded from 24 adult human inner ear specimens [25]. The reduced number of turns meanwhile observed at GW23 could also relate to inter-subject variation. Meanwhile, the ratio of the volume of the endolymphatic fluid in the cochlear duct to the length of the developing cochlear duct/ scala media remains directly proportional to the gestational age. The strategy we have pursued here using the centerline approach was helpful in deducing the impressive transformation of the cochlear duct during cochlear development.

Bredberg conducted the single measurements available on the developing human scala media [17]. He examined eleven human fetuses aged from GW12 to GW24 by using the block-surface technique followed by histological examination. The scala media in his specimens enlarged from 20 mm at GW12 to 39.60 mm at GW24 revealing an overall gain in length of the scala media. In our investigation the most prominent elevation in length was observed at GW19 when the hearing functionality is supposedly existent [12]. This sudden increase in length is evidenced due to closer examination of the specimens we have performed here using micro-CT. Previous research has used TSLIM based imaging and Amira® rendering software to visualize and identify characteristics of mouse inner ear development [26]. In the same study between embryonic day 14.5 and postnatal day 15 the scala media showed a three-fold increase in length (~ 2–6 mm) while the volume of the scala media enlarged by a factor of 10 (~ 0.025 μl–0.25 μl) [26]. Towards the completion of murine inner ear development process a slight decrease in volume, similar to our investigation on the human scala media, was described.

Although the inner ear exits the cell cycle by GW9 [31] and attains its anatomical landmarks like OHC, IHC and supporting hair cell formation by GW12, the inner ear morphology keeps continuously changing with gestational progression [32]. Previous studies performed using immune staining’s were able to visualize the development of the cochlear hair cells between GW 10 to 12 [8, 14]. We were also able to localize otic differentiation using transcription factors like Pax2 & Pax8 and chart the development of vestibular innervation using antibodies specific Beta III tubulin and peripherin during the same time period [7]. These and other morphological changes causes a volumetric increase in geometric size some of which are accompanied by the displacement of the sensory structures. Such displacement of the sensory structures causes a resultant cellular proliferation as well as by the ever growing distances between the centers of the different cristae and the centers of macular organs. In the specimens we examined the distance between the macular organs as well as the distance between the utricle and the posterior Crista ampullaris doubled in size, while the distance between the utricle to other cristae only slightly increases. This is indicative of the fact that the displacement of the sensory organs in the fetal vestibulum is uneven although the distance between the saccule to all the cristae increased uniformly. These increases in distance between the vestibular end organs are a consequence of its increasing size following its initial assemblage.

The development of the organ of Corti occurs at different time points along the spiral cochlear duct. Our histological analysis, including light and transmission electron microscopy, showed that there exists a gradient in development from the basal turn to the apical turn of the cochlea, with cells along the basal turn being more advanced in their development when compared to cells in the apical turn as evidenced from semi-thin sections from GW14. The cellular growth in the vestibulum is much more advanced towards this time point as implicit from the well-developed utricular hair cells, their stereocilia and surrounding supporting cells. These findings support previously described data of the fetal utricle at GW11 [7] and is an indicator of the early maturation of the vestibular organ. The support structures like the protocalyx, stereocilia and the kinocilia are an indicator of the early onset of the vestibular functionality.

The correlative approach previously described for studying bivalves [33] allow usage of micro-CT, light microscopy, and transmission electron microscopy for studying the same specimen. This allowed us to acquire structural information across various levels of biological organization. Analysis of gestational specimens started with the morphometric evaluation of the whole developing inner ear organ based on micro-CT volumes, while subsequently cellular and subcellular details were investigated. This provided new insights into human inner ear development, for which data has so far been scarce.

This is the first demonstrated usage of a big cohort of fetal specimens using histological techniques serial like semi-thin sectioning, ultra-thin sections, TEM and micro-CT in human fetal inner ear specimens. Previous reports by Fritzsch B using Scanning Thin-Sheet Laser Imaging Microscopy on prenatal and postnatal murine inner ear specimens had demonstrated a 3D analysis of optical sections [26]. 3D structures was obtained by SEM imaging where we could visualize the passage of an efferent nerve fiber through the not yet functional tunnel of Corti. The combinatorial approach we have pursued using micro-CT, light microscopy using serial semi-thin sections, TEM and SEM could give a broader outlook of selected regions.

## Conclusions

We observed changes in the structural, cellular and morphological aspects of the developing human inner ear including changes in ultrastructural organization with in the maturing sensory epithelia. Quantification of these volumetric and linear changes enabled us in realizing the complex developmental patterns of the human inner ear. Moreover, awareness of subcellular and cellular changes we observed in normal development could serve as a reference for better understanding both hearing and vestibular disorders.

## Methods

Eight human embryos and fetuses of varying gestational ages (GW11, GW12, GW14, GW15, GW19, GW23, GW33, and GW36) were utilized for this study. The specimens were assessed by trained anatomists and embryologists to ascertain that the specimens used in this study were devoid of any external or internal congenital defects. The age of the specimens was determined by evaluation of the Crown Rump Length (CRL) and foot length as well as other morphological parameters. Temporal bones from body donors were excised and fixed in Karnovsky’s formaldehyde-glutaraldehyde solution for several weeks. To ensure rapid fixative penetration, oval and round windows were penetrated with a needle, and the fixative gently perfused with a Pasteur pipette. Specimens were post-fixed in 2% osmium tetroxide for 2 to 10 h. After thorough washes in PBS, specimens were decalcified in EDTA, pH 7.2–7.4 for 2–3 weeks at 37^0^ C in a Milestone® HISTOS 5 microwave tissue processor. Following which the specimens were then thoroughly washed in PBS for two days. Subsequently, they were transferred to 50 and 70% ethanol 3 × 2 hours each and rotated on an overhead shaker (Heidolph® Reax) to remove the air bubbles.

### MicroCT image acquisition

Osmium-stained fetal temporal bones were mounted vertically in plastic syringes containing 70% ethanol [34]. MicroCT image acquisition was performed with an XRadia MicroXCT-400 (Carl Zeiss X-ray Microscopy, Pleasanton, CA, USA) at 45 kVp and 110 μA using the 0.4 X detector assembly. Projection images were recorded with an angular increment of 0.166° between projections. Depending on specimen contrast, exposure time was either 30 s or 60 s per projection image. Depending on specimen size, voxel resolution of reconstructed slices was ranging from 4.67 μm (smallest specimen, GW 11) and 14.50 μm (largest specimen, GW 36). Reconstructed image volumes were exported in DICOM format. Scans were imported to Amira® 6.4.0, and nerves and structures of the membranous labyrinth were manually segmented switching between the three orthogonal planes using the Segmentation editor. Segmented structures such as the membranous labyrinth, perilymphatic compartments of the whole inner ear, vestibular end organs, and vestibulocochlear nerve were visualized using volume and surface renderings.

### Scala media length measurement

MicroCT scans of the fetal human temporal bone belonging to all available gestational ages were manually segmented using Thermo Scientific™ Amira™ Software® v6.4.0 and validated independently by trained anatomists. Segmented labels were cropped to reduce file size. The scala media label was isolated using the Arithmetic tool [expression (“a==bundle number of scala media”)*1]. The result obtained was resampled by the factor 2 (Resample). Subsequently, the resampled volume was processed in the Segmentation editor using five voxel dilation (Grow selection) followed by three steps of contour smoothing (Smooth labels) and a five voxel erosion (Shrink selection). Based on the smoothed scala media mask, a skeletonization was performed (Centerline tree, number of parts = 1). The resulting spatial graph was smoothed (Smooth Line Set, Smoothing = 0.85, Adhere to original data = 0.05, Number of Iterations = 200). Finally, the length of the smoothed spatial graph representing the total length of the scala media was measured (Spatial Graph Statistics).

### Distances between the neurosensory regions

Sensory epithelia of the vestibular system from all available gestational stages were again manually segmented using Thermo Scientific™ Amira™ Software® v6.4.0. For all sensory epithelia, the center of mass was calculated using the Material Statistics tool. Using the X-, Y- and Z- coordinates of the region centers the distances between all neurosensory regions (3 Crista ampullaris and the macular organs of the utricle and the saccule) were measured as the vector magnitude representing the distance between two respective points (i.e., the distance between the centers of two neurosensory regions).

### Scala media volume

Volume measurement on different scala media labels where performed using the Amira™ Software® v6.4.0 *Material Statistics* function.

### Co-registration of micro CT and LM images

For two specimens (GW11 (refer Additional file 2), GW14), light microscopic images were registered into the micro CT volumes. For this, both the micro CT volume and selected light microscopic images from semi-thin sections were loaded into Amira® v6.4.0. For both modalities, masks for scala media, scala vestibuli, and scala tympani were created by manual segmentation. Next, the semi-thin section mask was manually pre-aligned into the micro CT volume mask using an *Orthoslice* with *Colorwash function* showing the overlay of the two segmentation masks. The *Register Images* tool was used for fine alignment of the semi-thin segmentation mask. Finally, transformation parameters were transferred to the original semi-thin section image.


**Additional file 2: Video S2.** Correlative approach demonstrating a three-dimensional rendering from a microCT scan of a 11 gestational week old fetal temporal bone registered onto a light microscopic image of a histological section from the very same specimen. (MPG 10043 kb)


### Correlative approach

After micro-CT imaging, all specimens were embedded and cut to correlate microCT with semi-thin sections and electron microscopic images (Handschuh et al., 2013).

### Tissue preparation for light (LM) and transmission Electron microscopy (TEM) analysis

Following micro-CT imaging fetal temporal bone samples were rinsed, dehydrated in graded ethanol series and embedded in EPON resin [35]. Serial sections were cut parallel to the central axis of the modiolus and stained with Toluidine Blue as described previously [36]. Images from 88 consecutive 1.5-μm-thick semi-thin sections were acquired with a TissueFAXS PLUS (TissueGnostics® Austria) system.

Ultrathin sections (90 nm) were cut on a Reichert Ultracut S microtome (Leica Microsystem, Wetzlar, Germany) with an ultra-diamond knife, mounted on dioxan-formvar-coated slot-grids (#G2500C, Christine Gröpl, Elektronenmikroskopie, Tulln, Austria) and stained 35 min with 0.5% (*w*/*v*) uranyl acetate, pH 4.4 and 10 min with 3% (w/v) lead citrate, pH 12 (Leica Ultrastainer, Leica Microsystem, Wetzlar, Germany). The ultrathin sections were examined using a Zeiss Libra 120.

### Scanning Electron microscopy

One cochlea was decalcified (Gestational age 13 weeks) in 0.1 M Na-EDTA, pH 7.4, for six weeks. Subsequently, they were washed in PBS, pH 7.4, dehydrated in graded ethanol (70, 80, 90, 95, and 100%; 10 min each), critical point dried, and attached to aluminum stubs. Specimens were coated in a BALTECH MED020 Coating System with gold-palladium to a nominal depth of 10–12 nm and viewed in a ZEISS DSM982 Gemini field emission electron microscope operating at 5 kV. Maximal resolution at this voltage was estimated to approximately 2 nm. Digital photos were taken at 1280–1024 ppi resolution.

### Statistics

All statistical analysis was performed using GraphPad Prism 7.1 software.

## Abbreviations

AT: Apical Turn
BT: Basal turn
CRL: Crown Rump Length
CS: Carnegie Stage
GER: Greater epithelial
GW: Gestational Week
IHC: Inner hair cells
KO: Kölliker’s organ
LER: Lower epithelial ridge
M: Modiolus
MicroCT: X-ray microtomography ridge
MT: Middle turn
OC: Organ of Corti
OHC: Outer hair cells
OtC: Otic Capsule
SCC: Semicircular canal
SM: Scala Media
ST: Scala Tympani
SV: Scala Vestibuli
TCF: Tunnel Crossing Fibers
TSB: Tunnel Spiral Bundles
